# Visual adaptation selective for individual limbs reveals hierarchical human body representation

**DOI:** 10.1167/jov.21.5.18

**Published:** 2021-05-18

**Authors:** Alexander Bratch, Yixiong Chen, Stephen A. Engel, Daniel J. Kersten

**Affiliations:** 1 Department of Psychology, University of Minnesota, Minneapolis, MN, USA; 2 Department of Biomedical Engineering, University of Minnesota, Minneapolis, MN, USA

**Keywords:** body perception, visual adaptation, hierarchical processing

## Abstract

The spatial relationships between body parts are a rich source of information for person perception, with even simple pairs of parts providing highly valuable information. Computation of these relationships would benefit from a hierarchical representation, where body parts are represented individually. We hypothesized that the human visual system makes use of such representations. To test this hypothesis, we used adaptation to determine whether observers were sensitive to changes in the length of one body part relative to another. Observers viewed forearm/upper arm pairs where the forearm had been either lengthened or shortened, judging the perceived length of the forearm. Observers then adapted to a variety of different stimuli (e.g., arms, objects, etc.) in different orientations and visual field locations. We found that following adaptation to distorted limbs, but not non-limb objects, observers experienced a shift in perceived forearm length. Furthermore, this effect partially transferred across different orientations and visual field locations. Taken together, these results suggest the effect arises in high level mechanisms specialized for specific body parts, providing evidence for a representation of bodies based on parts and their relationships.

## Introduction

The perception of human bodies is critical for social behavior (cf. Hu, Baragchizadeh & O'Toole, 2020). The determination of the spatial relationships between body parts, referred to here as pose estimation, is a particularly important source of information for a range of visual functions, including the recognition and interpretation of the actions of others. Because of its practical importance, the computational problem of pose estimation has recently received considerable attention (cf. Cao, Hidalgo, Simon, Wei, & Sheikh, 2019; Chen & Yuille, 2014). A key question has been how to represent body structure for efficient and robust pose computation. One approach is to represent body structure in terms of parts (e.g., hands, elbow, shoulder, etc.) constrained by their plausible spatial relationships (distance and angle). Visual computation then proceeds hierarchically first integrating low-level features into parts, then to part relationships, and finally to whole bodies (Chen & Yuille, 2014; Park, Nie & Zhu, 2018).

Human neuroimaging studies have provided evidence consistent with this hierarchical computation, specifically identifying distinct representations for body parts versus whole bodies. Studies of body selective cortical areas have demonstrated a cortical region sensitive to individual parts, but insensitive to their configuration/spatial relationships (the extrastriate body area [EBA]; Downing, Jiang, Shuman & Kanwisher, 2001). Conversely, research has found a region which is sensitive to the configuration of body parts as a whole, but not necessarily the individual parts themselves (the fusiform body areas [FBA]; Peelen & Downing, 2005). Such findings are consistent with the idea of an underlying neural mechanism for pose estimation based on a hierarchical organization of parts and relationships.

Recently, a number of investigations have used adaptation to explore perceptual representations of bodies. Adaptation, the process by which the visual system routinely updates its sensitivity to visual features, has been shown to operate at many levels of visual processing, from low-level features, such as orientation, spatial frequency, and color, to higher level properties, including viewpoint and shape, and class-specific attributes, such as inter-eye distance, identity, and gender of faces (cf. Blakemore & Campbell, 1969; Kaliukhovich & Vogels, 2016; Webster, 2015 for a review). Adaptation studies have also revealed interactions between lower and higher-level representations, possibly involving unidirectional and bidirectional signaling within the visual hierarchy (He, Kersten & Fang, 2012; Liu & Engel, 2020; Xu, Dayan, Lipkin & Qian, 2008). Although previous adaptation studies on body representation have revealed effects along high level dimensions, including gender (Ghuman, McDaniel & Martin, 2010; Kessler, Walls & Ghuman, 2013; Palumbo, D'Ascenzo & Tommasi, 2015; Palumbo, Laeng & Tommasi, 2013; Weigelt, Koldewyn & Doehrmann, 2010) as well as viewpoint (Lawson, Clifford & Calder, 2009), size, and weight (Ambroziak, Azañón & Longo, 2019; Brooks, Clifford, Stevenson, Mond, Stephen & Brooks, 2018; Glauert, Rhodes, Byrne, Fink & Grammer, 2009; Winkler & Rhodes, 2005), the hierarchical nature of body perception remains underexplored using adaptation.

Here, we investigated the hierarchical nature of body perception using adaptation. Specifically, we tested for the existence of distinct adaptable, high-level representations of individual body parts. We generated a set of forearm/upper arm limb pairs where the forearm had been lengthened or shortened. Observers viewed these arms and judged whether their forearms appeared too long or too short before and after adapting to arms, and other similar but semantically distinct objects, across different stimulus orientations and visual field locations. If adaptation arises from high level body part specific mechanisms, then it should transfer across visual field orientations and locations but not transfer to visually similar images of other body parts and non-body objects.

## Experiment 1

Neurons that represent high-level visual features have receptive fields whose responses are relatively invariant to low-level manipulations, such as retinal position and orientation (Gross, Bender & Rocha-Miranda, 1969). If visual adaptation causes changes in the response properties of such neurons, then effects of adaptation should transfer to different orientations. If, on the other hand, adaptation arises from neurons in early visual areas, where receptive fields are sharply tuned for orientation, then its effects should be relatively orientation-specific. The goal of Experiment 1 was to distinguish between these alternatives by measuring the extent to which adaptation to body parts transfers to other orientations in the visual field. Observers judged forearm length across two different orientations and subsequently adapted to a shortened forearm in one orientation. The shift in perceived forearm length was then assessed across the adapted and nonadapted orientations.

## Materials and methods

### Observers

Ten observers (7 men, and 3 women) participated in Experiment 1. All observers had normal or corrected to normal vision, were naive to the purpose of the experiment, and provided written informed consent. The experiment was approved by the Institutional Review Board of the University of Minnesota and procedures conformed to the Declaration of Helsinki.

### Apparatus

Stimuli were presented on a 24 inch NEC LCD display (resolution = 1920 × 1080 pixels; size = 52.7 × 29.6 cm; 55.6 × 33.0 degrees of visual angle from a viewing distance of 50 cm). A chin rest was used to maintain a constant viewing distance and head position. Stimuli were generated using MakeHuman version 1.1.1 and Blender version 2.79 and were presented within the Matlab programming environment (version R2016a) using in-house software and the Psychophysics Toolbox (Brainard, 1997).

### Stimuli

**Figure 1.**
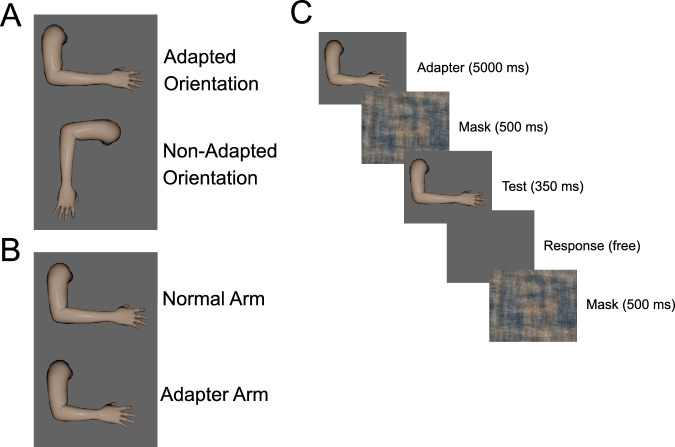
Overview of the stimuli and procedure used in Experiment 1. (**A**) The two orientations of the test stimuli. (**B**) Examples of the normal length forearm and 75% length forearm used as the adapter. (**C**) Procedure used for assessing adaptation in Experiment 1. Following an initial adaptation period, observers saw a “top-up” adapter image (5000 ms) prior to each test image (350 ms) and response period (free). The test image was flanked by a phase scrambled mask image (500 ms).

The images used for Experiment 1 were synthesized from an upper arm and forearm extracted from an average proportioned MakeHuman avatar with a skeletal rig. A custom rigging environment in Blender was used to generate individual images from this limb set. For all stimuli, the elbow joint of this limb set was positioned at approximately 90 degrees (see Figure 1).

To manipulate the relative proportions of the upper arm to the forearm (“physical arm length”), renderings were created by scaling the bone comprising the forearm (yielding a percent change of physical length). Seven total images were generated by setting the scale factor from 85% to 115% of veridical at 5% increments (7 total bins, shortest forearm = 9.4 degrees of visual angle; longest forearm = 12.0 degrees of visual angle). In Experiment 1, observers viewed these stimuli at two orientations: with the hand pointing to the right of the display (rightward, Figure 1a) or with the hand pointing to the bottom of the display (downward, Figure 1a). During adaptation, observers viewed an arm with a shortened forearm, where the bone had been scaled to 75% (forearm = 8.5 degrees of visual angle, Figure 1b). This adapter was only presented in the rightward orientation. All images were presented in color on a low luminance gray background.

### Procedure

Observers were tested individually in a dedicated testing room. Prior to the start of the experiment, observers were informed that an image of an arm would appear in the center of the display on each trial and that, on a given trial, the forearm would appear either lengthened or shortened. They were instructed to respond, using a computer keyboard, whether the presented arm on a given trial appeared to be “too long” or “too short.” Observers were allowed to move their eyes freely during the experiment.

A first block of trials measured perceived arm length in neutral, “baseline” conditions. An overview of the procedure can be seen in Figure 1c. On a given baseline trial, an arm image appeared in the center of the display for 350 ms, followed by a blank gray screen, during which observers indicated their response. After a response was collected, a mask consisting of a phase scrambled arm stimulus was presented for 500 ms prior to the start of the next trial. Baseline performance was assessed for both arm orientations in an intermixed block. Twenty-five trials were presented for each of the seven physical arm lengths at each orientation for a total of 350 trials.

A second block of trials measured perceived arm length following adaptation. Observers adapted by viewing the image of the arm with a shortened forearm, presented in the center of the display, for 5 minutes. As in baseline assessment, observers were allowed to move their eyes freely – they were not instructed to fixate. Perceived arm length was then measured while using a “top-up” paradigm to maintain adaptation. The trial structure was identical to baseline assessment, with the addition of a 5 second adapting image presentation followed by a 500 ms phase scrambled mask preceding the test stimulus presentation on each trial. As in the baseline block, 25 trials were presented for each physical arm length at each orientation, in a mixed block of 350 trials.

### Data analysis

For each observer, we first calculated the proportion of “too long” responses for each of the seven physical arm lengths. This was done independently for each orientation in both the baseline and post adaptation conditions. The resulting data was then fitted with a logistic function, enabling us to determine the point of subjective equality (PSE), defined as the physical forearm length estimated to produce “too long” responses on 50% of the trials.

**Figure 2.**
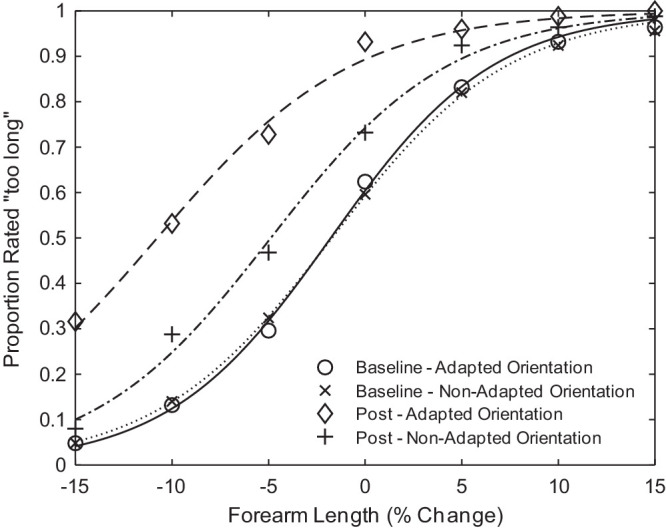
The average response rate and psychometric fits across observers (*N* = 10) in the baseline and post adaptation conditions in each orientation. The proportion of trials rated as “too long” is displayed as a function of forearm length.

The average response rate across observers and associated fits are shown in Figure 2. A leftward shift in the PSE on the physical arm length axis indicates an elevation in the number of “too long” responses, and thus indicates that the previously “normal” arm appeared too long following adaptation to the shortened forearm. Finally, we computed the shift in PSE as PSE at baseline minus PSE at post-test.

A linear mixed effects model, constructed within the R programming environment using the LMER package (Bates, Maechler, Bolker, Walker & Haubo Bojesen Christensen, 2015), was used to assess the results of Experiment 1. The model was constructed using the PSE values derived from the psychometric fits described in the previous paragraph. Fixed effects included adaptation (baseline versus post), orientation (adapted versus nonadapted), and the interaction between adaptation and orientation. Random effects included random slopes and intercepts for each individual observer. The fixed effects of the model were then analyzed using F-tests in the context of a type III ANOVA using the car package (Fox & Weisberg, 2019). Additionally, specific linear contrasts were analyzed with F-tests using the phia package (De Rosario-Martinez, 2015) and *p* value correction via the Holm-Bonferroni method.

## Results

To examine the impact of adaptation on perceived arm length, we measured the PSEs (forearm lengths that appeared “normal”) before and after exposure to the shortened forearm (see Figure 1 and Methods).

**Figure 3.**
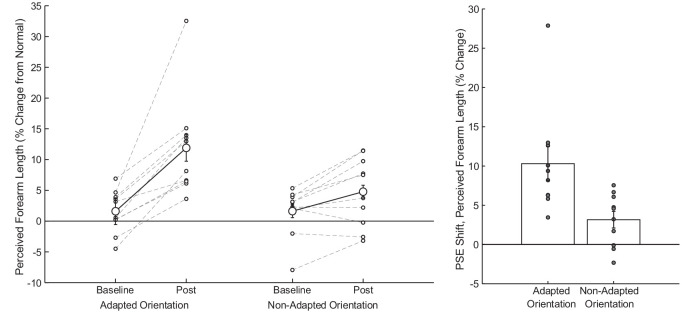
Results for Experiment 1. Left. Average (large, open circles connected by dark solid lines) and individual (small, open circles connected by dashed lines) PSEs in each orientation before and after adaptation. Right. PSE shifts (PSE at baseline minus PSE at post test) for each orientation, with individual data shown for each observer (small, gray circles). Error bars represent standard error of the mean (SEM).

Following adaptation to a shortened forearm, observers perceived test arms as being longer, and there was a trend for this effect to transfer across orientations. Figure 3 (left) displays PSEs for each individual observer (small gray circles connected by dashed lines) as well as the mean across observers (large open circle connected by solid lines). Figure 3 (right) summarizes these results in terms of the mean PSE shift from baseline for each orientation. For the adapted orientation, the perceived forearm length shifted by approximately 10%, whereas for the non-adapted orientation perceived forearm length shifted by approximately 3%. An ANOVA revealed significant main effects of adaptation (F[1, 9.43] = 21.07, *p* < 0.01) and orientation (F[1, 9.26] = 5.57, *p* < 0.05), as well as a significant interaction effect between adaptation and orientation (F[1, 18] = 19.88, *p* < 0.001). Planned contrasts confirmed a significant shift from baseline for the adapted orientation (F[1, 13.93] = 38.01, *p* < 0.001) and a trend in the nonadapted orientation (F[1, 13.93] = 3.58, *p* < 0.1), as well as a significant difference between the adapted and nonadapted PSE shifts (F[1, 9] = 19.88, *p* < 0.01).

## Experiment 2

The goal of Experiment 2 was to provide stronger evidence that adaptation to arm length could be attributed to higher level representations. Observers once again made judgments about forearm length but did so before and after adapting to three different adapter types, which varied between high and low level: an arm, a leg, and a pipe segment. To provide a more robust test for high-level representations, stimuli were presented mirrored about either side of a central fixation cross. If adaptation is based on relatively high-level mechanisms, as opposed to retinotopic-level mechanisms, then the effects should be at least partially invariant to change in arm orientation as a function of mirroring and to the overall change in retinotopic position. Furthermore, if the effect is in fact based on high-level mechanisms, the various adapter classes would enable us to determine if the effects arose from general object/shape mechanisms (in the case of transfer from the pipe adapter), general body processing mechanisms (in the case of transfer from the leg adapter), or limb specific mechanisms (in the case of transfer only from the arm adapter).

## Materials and methods

### Observers

Twenty-four observers (9 men, and 15 women) participated in Experiment 2. All observers had normal or corrected to normal vision, were naive to the purpose of the experiment, and provided written consent. The experiment was approved by the Institutional Review Board of the University of Minnesota and procedures conformed to the Declaration of Helsinki.

### Apparatus

Stimuli were presented on the same monitor used in Experiment 1, but with the viewing distance changed to 60 cm (size = 52.7 × 29.6 cm; 47.4 × 27.7 degrees of visual angle). A chin rest was used to maintain a constant viewing distance. Stimulus generation and presentation was performed using the same methods in Experiment 1.

### Stimuli

**Figure 4.**
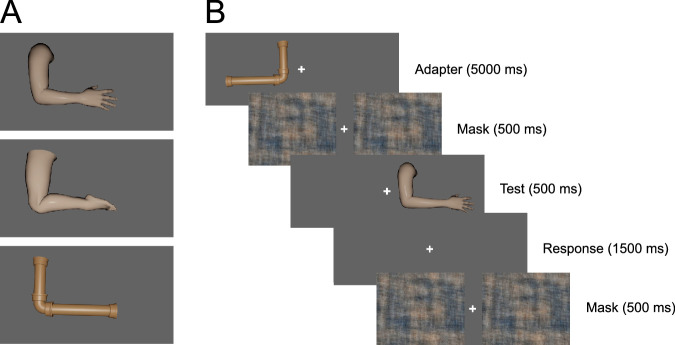
Overview of the stimuli and procedure used in Experiment 2. (**A**) The three adapter types (arm, leg, and pipe) used in Experiment 2. (**B**) Procedure used for assessing adaptation in Experiment 2. Adapter images were presented in one hemifield (5000 ms) prior to each test trial (500 ms) and response period (1500 ms). The test image was flanked by a phase scrambled mask image (500 ms).

Experiment 2 used the same arm images as Experiment 1 (shortest = 7.8 degrees of visual angle; longest = 10 degrees of visual angle). However, whereas a free-viewing paradigm was used in Experiment 1, stimuli in Experiment 2 were presented on either side of a central fixation cross (Figure 4b).

In addition to the shortened arm adapter used in Experiment 1, we used two additional adapters: a shortened leg and a copper pipe (Figure 4a). The shortened leg adapter was generated using the same procedure as the arm adapter; the lower leg bone was scaled to 75% of its original length. Additionally, the rendering camera distance was adjusted such that the leg subtended approximately the same visual angle as the shortened arm adapter. The copper pipe adapter was created such that it had similar mid-level geometric properties (same angular size and part length ratios) to the arm and leg adapters, but with small shape differences (e.g., pipe joints) consistent with a very different high-level semantic category. All adapters subtended approximately 7.1 degrees of the visual angle in length.

### Procedure

Each observer participated in three sessions. Each session was similar to those in Experiment 1, but used a different adapter (arm, leg, or pipe). Order of the adapter was counterbalanced across observers. The adapter in this experiment was presented either to the left or right of fixation, and this factor was also counterbalanced across observers.

Task and response instructions were the same as in Experiment 1, with four main differences: (1) observers were instructed to maintain fixation on the central cross throughout the duration of the experiment; (2) observers were given a fixed duration 1500 ms interval in which to respond before the mask image was presented at the beginning of the next trial; (3) test images and adapters were shifted away from the central cross, either to the left or right, such that the edge of the stimulus was approximately 0.5 degrees away from the cross center; and (4) there was no initial adaptation period. Adapting images were presented only in the 5 second “top-up” intervals at the start of each trial. Additionally, prior to beginning the baseline portion of the experiment on the first session, observers were familiarized with the stimulus set and the presentation paradigm. Observers also completed 50 practice trials at the start of their first session.

Baseline perception of arm length was measured at the start of each session, in a block of 210 trials containing 15 trials at each of the seven physical arm lengths presented in each hemifield. Trial order within this 210 trial block was randomized. A second 210 trial block in each session then measured perceived arm length in the presence of an adapter.

### Data analysis

Quality metrics were used in Experiment 2 to assess the viability of data for each observer. Specifically, an observer who responded to less than 90% of trials or whose psychometric function fits produced a slope less than or equal to 0 were excluded from the final analysis. Of the 24 observers who participated in Experiment 2, 22 observers met the predefined data quality criteria and were entered into the analysis.

**Figure 5.**
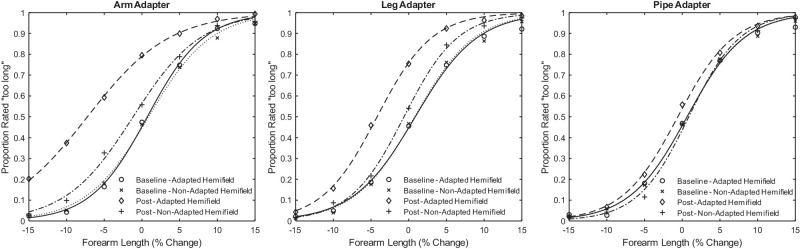
The average response rate and psychometric fits across observers in the baseline and post adaptation conditions in each hemifield for each adapter type. The proportion of trials rated as “too long” is displayed as a function of forearm length.

The analysis of Experiment 2 used the same methods and software packages as Experiment 1. In short, the 50% PSEs were derived from logistic fits (see Figure 5), entered into a linear mixed effects model, and analyzed using an ANOVA and F-test linear contrasts. Fixed effects included adaptation (baseline versus post), hemifield (adapted versus nonadapted), and adapter (arm versus leg versus pipe), and random effects included random slopes and intercepts for each individual observer.

## Results

**Figure 6.**
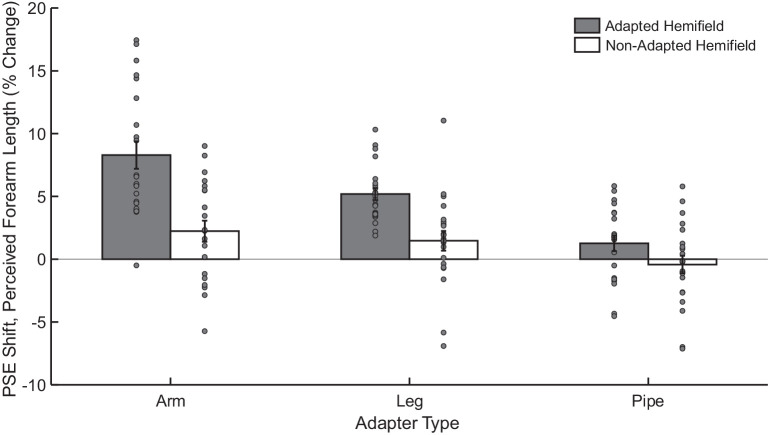
Results of Experiment 2. The average PSE shift (PSE at baseline minus PSE at post test) is shown for each adapter type in the adapted (gray bar) and nonadapted (white bar) hemifields. Datapoints for individual observers are shown as gray dots. Error bars represent SEM.

As in Experiment 1, we assessed adaptation by comparing the PSEs when perceiving arm length before and after adaptation. This was assessed across the three adapter types (shortened arm, shortened leg, and pipe) and both hemifields (adapted and nonadapted). Figure 6 summarizes these results in terms of the mean PSE shift from baseline for each adapter in each hemifield.

Adaptation was strongest for the arm adapter, intermediate for the leg adapter, and weakest for the pipe adaptor, and in all cases was stronger for the adapted hemifield than the nonadapted hemifield. The ANOVA revealed significant main effects of adaptation (F[1, 21.00] = 38.47, *p* < 0.001), hemifield (F[1, 21.01] = 5.13, *p* < 0.05), and adapter type (F[2, 21.00] = 4.50, *p* < 0.05). Furthermore, there were significant two-way interactions between hemifield and adaptation (F[1, 146.99] = 55.47, *p* < 0.001), adaptation and adapter (F[1, 146.99] = 30.10, *p* < 0.001), and hemifield and adapter (F[1, 146.99] = 7.30, *p* < 0.001), as well as a significant three-way interaction between hemifield, adaptation, and adapter type (F[1, 146.99] = 6.07, *p* < 0.01).

When adapting to a shortened forearm, observers perceived test arms in both the adapted hemifield and nonadapted hemifield as being significantly longer (adapted PSE shift = 8.29, F[1, 94.42] = 121.97, *p* < 0.001); nonadapted PSE shift = 2.24, F[1, 94.42] = 8.86, *p* < 0.05). Adaptation was greater in the adapted than nonadapted hemifields (PSE shift difference = 6.06, F[1, 147] = 46.51, *p* < 0.001).

Adaptation from viewing a shortened leg produced a markedly smaller effect on the arm in the adapted hemifield than when adapting to an arm (PSE shift difference = 3.11, F[1, 147] = 12.22, *p* < 0.01), although the former was significantly above zero within the adapted hemifield (PSE shift = 5.19, F[1, 94.42] = 47.73, *p* < 0.001). Contrary to the arm adapter, observers did not perceive arms as significantly longer in the nonadapted hemifield following adaptation to the shortened leg (1.47% PSE shift, F[1, 94.42] = 3.84, *p* = 0.16), and this shift was not significantly different from the nonadapted hemifield effect seen with the arm adapter (0.77% PSE shift difference, F[1, 147] = 0.74, *p* = 0.39). As with the arm, the effect was significantly larger in the adapted versus nonadapted hemifield (PSE shift difference = 3.72, F[1, 147] = 17.51, *p* < 0.001).

Finally, when adapting to the pipe, observers experienced no significant change in their perceived arm length in either the adapted hemifield (1.27 PSE shift, F[1, 94.42] = 2.84, *p* = 0.19) or in the nonadapted hemifield (-0.42 PSE shift, F[1, 94.42] = 0.31, *p* = 0.58), though there was a trend between the two hemifields (1.69 PSE shift difference, F[1, 147] = 3.60, *p* < 0.1). The observed effects were significantly lower than the effect for the arm adapter in both the adapted hemifield (7.03 PSE shift difference, F[1, 147] = 62.60, *p* < 0.001) and nonadapted hemifield (2.66 PSE shift difference, F[1, 147] = 8.94, *p* < 0.01). The effect was also significantly lower than for the leg adapter in the adapted hemifield (3.92 PSE shift difference, F[1, 147] = 19.48, *p* < 0.001) and trend in the nonadapted hemifield (1.89 PSE shift difference, F[1, 147] = 4.53, *p* < 0.1).

We also constructed an additional model, including hemifield of adaptation (left versus right). Here, we observed a trend for a main effect of hemifield of adaptation (F(1, 139.99] = 3.39, *p* < 0.1), but no associated interactions. Supplementary Figure S1 shows division of the effects as a function of hemifield of adaptation. Given prior literature of lateralization of body selective cortex (Downing, Jiang, Shuman & Kanwisher, 2001; Peelen & Downing, 2005; Willems, Peelen & Hagoort, 2010), future work is needed to investigate how this plays a role in these adaptation effects.

## Discussion

The goal of the present study was to test for distinct high-level representations of body parts. The results across both experiments are consistent with high-level, body-part specific mechanisms that could be used to compute spatial relationship between parts.

Our key prediction was that adaptation should transfer across features for which higher-level neurons are less well-tuned and lower-level neurons are more tightly tuned, specifically retinal orientation and location. In Experiment 1, we found that when adapting to shortened forearms, observers perceived forearms as significantly longer in the adapted orientation. A trend in this direction was also observed in the nonadapted orientation, albeit with a significantly reduced magnitude relative to the adapted orientation. This effect was strengthened in reliability in Experiment 2; a significant adaptation effect was measured in both the adapted and nonadapted visual hemifield when observers adapted to a shortened forearm (once again, with a reduction in magnitude in the nonadapted hemifield). Together, these findings suggest that the effect is, at least in part, arising from nonretinotopic, high-level neurons.

Another prediction was that adaptation should be greater for the same body part as the adapter than for other visually similar body parts and objects. Experiment 2 revealed that when observers adapted to legs with shortened lower legs, they once again perceived arms as longer, but the magnitude of the effect was reduced relative to arms in the adapted hemifield, and not significant in the nonadapted hemifield. In addition, no significant shifts in perception were found when observers adapted to an image of a pipe with an aspect ratio matched to the shortened forearm and leg adapters. Such a result suggests that not only does the effect observed in both Experiments 1 and 2 appear to have a high level component, but that the effect can even be differentiated at the level of specific body parts, indicating there may be underlying mechanisms devoted to the processing of limb subsets.

Our findings and interpretations, specifically with respect to lower level, retinotopic phenomena, are consistent with other work on body adaptation, which has mainly focused on whole body adaptation effects. Past findings have shown that, for instance, adaptation to body size/weight could not be explained simply by adaptation to basic shapes of a similar aspect ratio (Hummel, Grabhorn & Hohr, 2012). Furthermore, these types of adaptation effects appear to transfer to different viewpoints as well as to different body poses (Sekunova, Black, Parkinson & Barton, 2013), suggesting the engagement of body-specific mechanisms rather than low-level, retinotopic mechanisms.

Prior work has also demonstrated that body size/weight effects transfers across identity (Hummel, Rudolf, Brandi, Untch, Grabhorn, Hampel & Mohr, 2012). Such a finding helps to potentially elucidate a general location of these mechanisms within the visual hierarchy. Although perceptual effects pertaining to basic body shape and size likely arise from a high level, body specific mechanisms, such mechanisms are likely distinct from and exist earlier in the visual hierarchy than mechanisms which process identity.

Consistent with this idea, recent evidence in the face perception domain has found that in both humans (Grill-Spector, Weiner, Kay & Gomez, 2017; Tsao, Moeller & Freiwald, 2008) and non-human primates (Freiwald & Tsao, 2010; Meyers, Borzello, Freiwald & Tsao, 2015), sensitivity to identity appears to increase throughout the progression in the visual hierarchy, peaking near the top (e.g., anterior temporal lobe), whereas sensitivity to basic within-category part relationships peaks earlier on (e.g., medial temporal lobe). In line with these intuitions on hierarchical location, functional magnetic resonance imaging (fMRI) investigations of the body size/weight adaptation effects have further revealed that higher level, body-selective visual cortical regions (e.g., EBA and FBA) but not lower level regions (e.g., V1) nor very high level regions (e.g., anterior temporal) appear to be involved with body size/weight adaptation (Hummel, Rudolf, Brandi, Untch, Grabhorn, Hampel & Mohr, 2013).

However, our work not only suggests an engagement of body-specific mechanisms but suggests one which appears to be sensitive to specific body parts. Consistent with our findings, as well as the fMRI findings noted above, recent studies have been able to further elucidate the level of representation of body parts within body selective regions of visual cortex. Using fMRI, it has been demonstrated that there are regions within lateral occipital cortex that have selective responsiveness to individual body parts (Orlov, Makin & Zohary, 2010). Furthermore, it has been shown that neural activity within body selective visual cortical areas yields distinct patterns of activity for individual body parts, suggesting fine grain representation for individual body parts within body selective cortex (Bracci, Caramazza & Peelen, 2015).

## Conclusions

Our results provide further evidence of specific high level representations for body stimuli and argue for a hierarchical arrangement in the representation of this stimulus class. The effects of adaptation appear to not only rely on representations specific to body stimuli, but mechanisms which are sensitive to the spatial relationships between individual parts. Future work should be able to provide further detail into the specificity of this representation, as well as investigate the neural basis of the effect and its consistency with recent findings in the fMRI literature.

## Supplementary Material

Supplement 1
