# Supplementary material for: Visual adaptation selective for individual limbs reveals hierarchical human body representation
Source: J Vis. 2021 May 18;21(5):18. doi: 10.1167/jov.21.5.18 (PMC8142707; doi:10.1167/jov.21.5.18)
Supplement: Supplement 1 [file jovi-21-5-18_s001.docx]

**Supplementary Material**

**
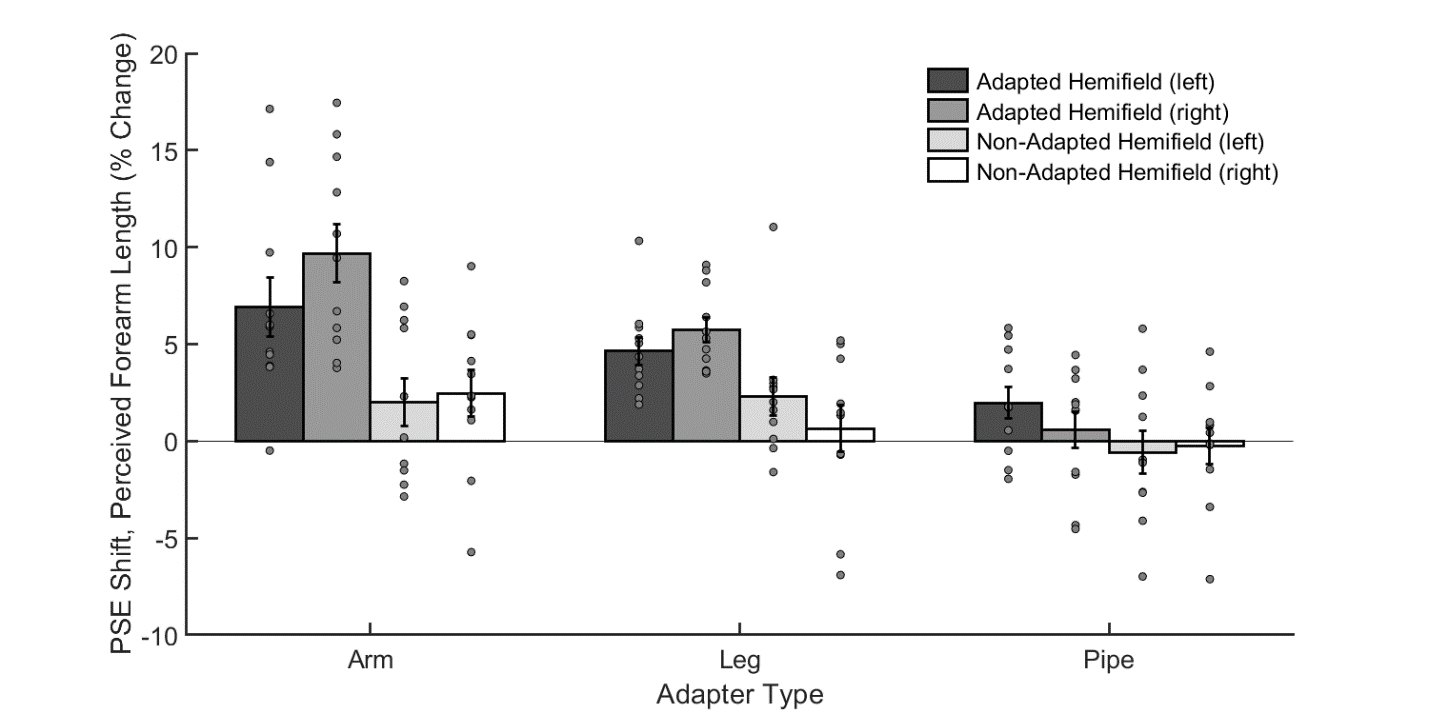
**

**Supplementary Figure 1.** Results of Experiment 2 when divided by hemifield of adaptation (left vs right). The average PSE shift (PSE at Post-test minus PSE at Baseline) is shown for each adapter type, in the adapted and non-adapted hemifields, and as a function of hemifield in which each observer adapted (left vs right). Datapoints for individual observers are shown as gray dots. Error bars represent SEM.
